# Knowledge‐based planning for the radiation therapy treatment plan quality assurance for patients with head and neck cancer

**DOI:** 10.1002/acm2.13614

**Published:** 2022-04-30

**Authors:** Wenhua Cao, Mary Gronberg, Adenike Olanrewaju, Thomas Whitaker, Karen Hoffman, Carlos Cardenas, Adam Garden, Heath Skinner, Beth Beadle, Laurence Court

**Affiliations:** ^1^ Department of Radiation Physics The University of Texas MD Anderson Cancer Center Houston Texas USA; ^2^ UTHealth Graduate School of Biomedical Sciences The University of Texas MD Anderson Cancer Center Houston Texas USA; ^3^ Department of Radiation Oncology The University of Texas MD Anderson Cancer Center Houston Texas USA; ^4^ Department of Radiation Oncology The University of Alabama at Birmingham Birmingham Alabama USA; ^5^ Department of Radiation Oncology University of Pittsburgh Pittsburgh Pennsylvania USA; ^6^ Department of Radiation Oncology Stanford University Stanford California USA

**Keywords:** knowledge‐based planning, head and neck cancer, quality assurance

## Abstract

This study aimed to investigate the feasibility of using a knowledge‐based planning technique to detect poor quality VMAT plans for patients with head and neck cancer. We created two dose–volume histogram (DVH) prediction models using a commercial knowledge‐based planning system (RapidPlan, Varian Medical Systems, Palo Alto, CA) from plans generated by manual planning (MP) and automated planning (AP) approaches. DVHs were predicted for evaluation cohort 1 (EC1) of 25 patients and compared with achieved DVHs of MP and AP plans to evaluate prediction accuracy. Additionally, we predicted DVHs for evaluation cohort 2 (EC2) of 25 patients for which we intentionally generated plans with suboptimal normal tissue sparing while satisfying dose–volume limits of standard practice. Three radiation oncologists reviewed these plans without seeing the DVH predictions. We found that predicted DVH ranges (upper–lower predictions) were consistently wider for the MP model than for the AP model for all normal structures. The average ranges of mean dose predictions among all structures was 9.7 Gy (MP model) and 3.4 Gy (AP model) for EC1 patients. RapidPlan models identified 7 MP plans as outliers according to mean dose or *D*1% for at least one structure, while none of AP plans were flagged. For EC2 patients, 22 suboptimal plans were identified by prediction. While re‐generated AP plans validated that these suboptimal plans could be improved, 40 out of 45 structures with predicted poor sparing were also identified by oncologist reviews as requiring additional planning to improve sparing in the clinical setting. Our study shows that knowledge‐based DVH prediction models can be sufficiently accurate for plan quality assurance purposes. A prediction model built by a small cohort automatically‐generated plans was effective in detecting suboptimal plans. Such tools have potential to assist the plan quality assurance workflow for individual patients in the clinic.

## 1 INTRODUCTION

Knowledge‐based planning (KBP) and automated planning techniques have been extensively explored to tackle the challenge of inconsistent and inefficient treatment planning in radiation therapy.^1^ KBP typically uses a library of plans from previously treated patients as a knowledge base and develops models associating geometric features and corresponding dosimetry from those plans to predict possibly achievable dosimetry for a new patient.^2^, ^3^ The objectives of plan optimization are then created based on the predicted dosimetry, followed by manual or automatic adjustment of those objectives in multiple rounds until desired dosimetric measures are achieved and the optimal plan is generated.^4^, ^5^ In addition to active research on KBP techniques, KBP‐based tools have been introduced in commercial treatment planning systems (TPS), including Eclipse (Varian Medical Systems, Palo Alto, CA),^6^, ^7^, ^8^ RayStation (RaySearch Laboratories AB, Stockholm, Sweden)^9^, ^10^ and Pinnacle^11^ (Philips Healthcare, Fitchburg, WI)),^12^ to enhance consistency and efficiency in planning and improve plan quality in clinical practice.

Recent reports have shown promising results when using KBP solutions in routine clinical planning and clinical trials.^6^, ^13^, ^14^, ^15^, ^16^, ^17^ As an automated tool capable of providing dose predictions and recommending optimization objectives for individual patients, KBP may guide the plan optimization process to achieve high‐quality plans more efficiently than the conventional approach, which is entirely based on trial and error. Therefore, current studies on KBP primarily focus on its utility in plan generation.^18^, ^19^, ^20^ However, implementing a KBP solution, whether in‐house or commercial, for plan generation still requires a certain amount of customization, including how to train the prediction model and how to optimize the plan. Even with a given prediction model, a multi‐round optimization process with intelligent (i.e., manual) intervention is needed, as a single round of optimization using automatically generated objectives typically is unable to produce a clinically acceptable plan. While such customization gives clinics flexibility to tailor plans for their own clinical practices and goals, it also can lead to inconsistent performances and hinder its adoption and potential gain in efficiency.

There are other potential applications of KBP in the clinical practice of radiation therapy, including plan quality assurance (QA) without user customization of optimization steps. Specifically, a selected cohort of patients with plans with consistent and high‐quality dosimetry could be used to build a prediction model with KBP, and the resulting model could be used as an independent QA tool to automatically detect suboptimal plans for new patients. Potential advantages of KBP‐based QA include minimum manual intervention, low barrier for clinical adoption, little interruption to the clinical workflow, and assuring consistent plan quality for clinical trials. Unlike dose–volume histogram (DVH)‐based checklists, which can flag unsafe plans, KBP‐based QA could potentially be used to flag suboptimal plans—that is, plans that could be further improved. Several groups have reported evaluations of in‐house and commercial DVH prediction models for organs at risk (OARs) in intensity‐modulated radiation therapy (IMRT) planning,^3^, ^16^, ^21^, ^22^, ^23^, ^24^ and Tol et al.^25^ evaluated the performance of using RapidPlan, a KBP solution available in the Eclipse TPS, for volumetric arc therapy (VMAT) plan QA. Each of these studies used plans with consistent dosimetric quality to both train and evaluate the prediction models. They all found that accurate DVH prediction could be achieved with these models.

In the present study, we mainly investigate (1) the effectiveness of using KBP trained by consistent and high‐quality automated plans to detect suboptimal plans; and (2) the potential impact on physician plan review in clinical practice. We aimed to provide a preclinical validation of using RapidPlan DVH prediction for VMAT plan QA for patients with head and neck cancer. Four unique aspects of this work include:

- One DVH prediction model were trained by a small cohort of patients with consistent and high‐quality automated plans;
- One evaluation cohort had clinically acceptable plans, and plans in the other cohort were intentionally generated to have suboptimal normal tissue sparing;
- Besides anatomical structures, non‐anatomical structures to control normal sparing were also evaluated;
- Plans were reviewed by multiple radiation oncologists from different institutions.

## 2 METHODS

### 2.1 Creating DVH prediction models

For model generation and evaluation, we selected 50 patients with head and neck cancer who had been treated with VMAT at our institution. This analysis was performed with Institutional Review Board approval. The cohort included patients with a variety of primary tumor locations, including cancers of the oropharynx, nasopharynx, hypopharynx, larynx, and oral cavity. Geometrical data included the original clinically utilized targets, OARs, and planning structures; there were up to three separate planning target volumes (PTVs) drawn by physicians with different dose levels. The planning of clinical plans for these patients had involved 10 dosimetrists at our clinic and the assignments of cases were random given each dosimetrist's schedule and workload. The plans were created based on our working planning protocol and approved by attending physicians. In addition to the clinical plan used in treatment, an automated plan was generated for each patient using a fully automated TPS, the RadiationPlanning Assistant (RPA).^26^, ^27^, ^28^, ^29^, ^30^ The RPA is able to generate clinically acceptable VMAT plans with high consistency for patients with head and neck cancer; additional details of the planning process have been published.^31^

We used 25 patients from the 50‐patient cohort to form a training cohort for training the DVH prediction model in RapidPlan (Varian Medical Systems, Palo Alto, CA). The other 25 patients formed evaluation cohort 1 (EC1) to assess model prediction accuracy (see Table 1). Two separate RapidPlan models were created according to two different training libraries, that is, manual plans (clinical plans created manually by planners’ or physicians’ intervention and used for treatment) and automated plans (automatically generated by the RPA without intervention or modification; also reviewed and deemed clinically acceptable by a radiation oncologist). We will call these two models the manual planning (MP) and automated planning (AP) models, respectively.

**TABLE 1:** Clinical characteristics of training and evaluation cohorts of head and neck cancer patients; 25 patients in each cohort

	Training cohort		Evaluation cohort 1		Evaluation cohort 2	
Primary tumor site	Number of patients	PTV dose, Gy* median (range)	Number of patients	PTV dose, Gy* median (range)	Number of patients	PTV dose, Gy* median (range)
Oropharynx	10	69.96 (60–70)	11	69.96 (60–70)	9	69.96 (69.96–70)
Nasopharynx	5	60 (60–66)	5	60 (60–66)	2	69.96 (69.96–70)
Hypopharynx	2	70 (70–70)	4	68 (60–68)	1	70 (70–70)
Oral cavity	5	60 (60–70)	4	60 (60–70)	9	60 (60–69.96)
Larynx	3	70 (66–70)	1	70 (70–70)	4	70 (60–70)

* PTV dose indicates the highest prescribed dose for a PTV.

RapidPlan in the eclipse TPS is configured to build a model that correlates the geometric relationships between PTVs and OARs with DVHs of the input training plans by principal component analysis and regression analysis. After the model is trained, that is, after the model parameters are determined, it can predict DVHs of matched OARs for a new patient anatomy. Both the MP and AP models included the following OARs: left parotid, right parotid, larynx, oral cavity, esophagus, mandible, brainstem, and spinal cord. Mean doses and goodness‐of‐fit model parameters *R* ^2^ for these 8 OARs are listed in Table 2. The average *R* ^2^ values [range] are 0.680 [0.405, 0.878] for the MP model and 0.886 [0.630, 0.961] for the AP model. Furthermore, for the AP model, two non‐anatomical structures that are used in the RPA automatic planning, that is, a posterior neck (PostNeck_Avoid) and pharyngeal airway plus cervical vertebrae (Airway_Avoid) were also included (see Figure S1 in the Supporting information for examples). These two structures were automatically generated by the autocontouring tools within the RPA in order to assist plan optimization for controlling hotspots in normal tissues located in the posterior area of the neck and approximal region around the vertebral column (e.g., the vertebral column adding a 5‐mm margin and subtracting all PTVs). The *R* ^2^ values were 0.834 and 0.856 for PostNeck_Avoid and Airway_Avoid models, respectively.

**TABLE 2:** Mean doses and model parameters *R* ^2^ of both manual planning (MP) and automated planning (AP) RapidPlan models for eight organs at risk (OARs)

		Goodness of fit: *R* ^2^	
Structure	Mean dose (Gy)	MP model	AP model
Left parotid	25.2±13.2	0.405	0.939
Right parotid	26.1±9.7	0.405	0.939
Larynx	53.5±12.6	0.659	0.943
Oral cavity	37.8±9.5	0.877	0.961
Mandible	38.9±8.4	0.803	0.849
Esophagus	30.9±10.9	0.878	0.883
Brainstem	16.2±9.9	0.836	0.940
Spinal cord	26.0±3.8	0.577	0.630

### 2.2 Evaluating prediction accuracy

To assess the prediction accuracy of the two RapidPlan models, we used an evaluation cohort of 25 patients (EC1 in Table 1). Plans used in model training and evaluation were generated by the same planning approach, that is, either manual or automated planning for the MP or AP models, respectively. RapidPlan can predict a range (mean ± 1 standard deviation) of DVHs for a selected OAR. These predicted DVHs of all OARs included in the RapidPlan model were compared with the achieved DVHs of the manual and automated plans for EC1 patients. Importantly, target coverage goals were met for all plans, as the same dosimetric requirements for the target were guaranteed during plan generation, for example, at least 95% of the high‐dose PTV receiving no less than 95% of the prescribed dose, no more than 1 cm^3^ of the high‐dose PTV receiving more than 107% of the prescribed dose, etc. Of note, RapidPlan does not predict DVH for target volumes.

### 2.3 Plan quality assurance and physician review

We evaluated a cohort of another 25 patients (EC2 in Table 1) that were unseen by the RapidPlan models. Two dosimetrists were tasked with generating manual plans with suboptimal normal tissue sparing for this cohort. The test plans were optimized such that their target coverage and OAR sparing met the dosimetric requirements of our clinical protocols, but additional OAR sparing could be achieved (automated plans were also generated for those patients as the benchmark for comparing OAR sparing with the test plans; details will be discussed in Section 3). No interaction between dosimetrists and physicians took place. In other words, these plans had not gone through a comprehensive validation and iterative review as for clinical plans, so they could mimic plans that are suboptimal but not in very poor quality as outliers seen in practical practice. These plans from EC2 were used to assess the effectiveness of KBP as a QA tool when the training and evaluation plans are generated by different planning approaches, unlike plans from EC1.

The AP model was used to predict DVHs for these manual test plans. The AP model was selected over the MP model for two main reasons: (1) the AP RapidPlan model may be more precise than the MP model because the plan quality is more consistent for automated plans than manual plans based on our experience^31^; and (2) the AP model includes additional non‐anatomical planning structures to assess more normal tissues than the MP model.

To assess the clinical acceptability of these plans, three radiation oncologists specializing in treating head and neck cancer from three different institutions reviewed the dosimetric quality of the test plans without seeing the DVH predictions. In a free‐form review, each oncologist examined slice‐by‐slice dose distributions, DVH curves, and dose statistics, and then provided their judgment as to whether sparing of each of the structures included in the RapidPlan model needed improvement. We then analyzed the degree of agreement between physician reviews and model predictions.

## 3 RESULTS

### 3.1 Prediction accuracy evaluation of the MP and AP models

The trained RapidPlan models generated a range of DVHs between the upper and lower prediction, that is, mean ± 1 standard deviation, for each patient in EC1. The ranges (upper – lower predictions) were consistently wider for the MP model predictions than for the AP model for all normal structures. For the 25 patients in EC1, the average range of mean dose predictions among all structures were 9.7 Gy (ranging from 6.1 to 14.0 Gy) for the MP model, and 3.4 Gy (ranging from 1.6 to 5.9 Gy) for the AP model. Detailed data can be found in Table 3. Larger ranges between upper and lower predictions resulted in the MP model than the AP model reflects higher variability in OAR sparing of MP plans used in model training compared to that of AP plans for all structures.

**TABLE 3:** Mean, minimum, and maximum of predicted range (upper–Lower prediction) of mean or *D*1% dose in Gy for different organs at risk (OARs) among 25 patients in evaluation cohort 1

	MP model		AP model	
Structure	Mean	Min, Max	Mean	Min, Max
Left parotid (*D*_mean_)	13.2	3.8, 16.1	3.0	1.7, 5.4
Right parotid (*D*_mean_)	14.0	10.3, 16.3	3.2	2.3, 5.4
Larynx (*D*_mean_)	8.7	2.1, 12	5.0	2.7, 7.8
Oral cavity (*D*_mean_)	6.1	1.0, 9.9	2.6	1.5, 3.2
Mandible (*D*_mean_)	7.2	2.8, 10	5.9	3.2, 7.5
Esophagus (*D*_mean_)	9.0	1.0, 19.7	3.5	0.5, 5.2
Brainstem (*D*_1%_)	12.4	3.2, 17.7	1.6	0.0, 3.6
Spinal cord (*D*_1%_)	6.8	0.2, 14.2	2.7	0.0, 8.8

Abbreviations: AP, automated planning; MP, manual planning.

**FIGURE 1:**
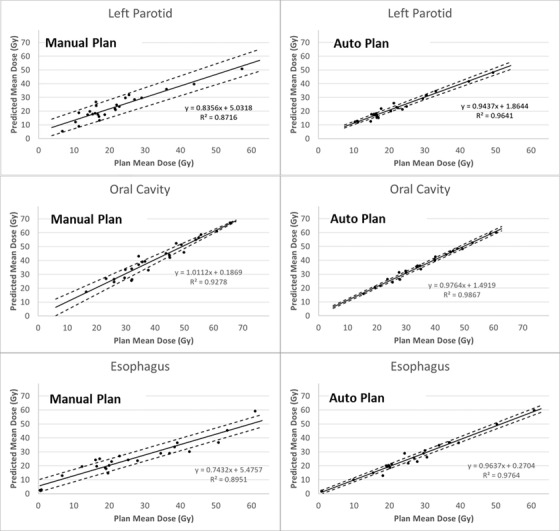
Linear regression between achieved and predicted mean doses to the left parotid, oral cavity, and esophagus for the 25 patients in evaluation cohort 1. Solid lines are fitted by the achieved mean dose of manual or automated plans and the predicted mean dose (derived from the average of upper and lower predicted dose–volume histograms (DVHs) by RapidPlan) of the manual planning (MP) or automated planning (AP) models. Dashed lines indicate regressions using the upper and lower predictions

The average of the upper and lower predicted DVHs was computed to evaluate the prediction accuracy for EC1 patients. Figure 1 shows the comparison of predicted and achieved mean doses for the left parotid, oral cavity, and esophagus, as examples. Results for other OARs are included in Figure S2. Note that the MP and AP models were evaluated by achieved manual and automated plans, respectively. For all OARs, predicted mean doses showed a linear relationship with the achieved mean doses for both models. The average of *R* ^2^ from the linear regressions for different OARs was 0.86 for the MP model and 0.97 for the AP model. This indicated that the AP model was more precise in predicting OAR mean doses than the MP model. Slopes of the linear fits were almost all less than 1, meaning that both models might overpredict achievable mean doses, except the MP model for the oral cavity, which had a slope slightly higher than 1.

To establish a threshold of predicted dose that could identify suboptimal OAR sparing, the difference between the predicted and achieved doses was first analyzed. We compared the upper prediction from the RapidPlan models with the achieved dose in order to account for model errors and avoid overprediction (Figure 1). Mean dose was used to evaluate parallel organs including parotids, larynx, and oral cavity, and *D*1% (dose received by 1% of a structure) was used to evaluated serial organs including brainstem and spinal cord.

**FIGURE 2:**
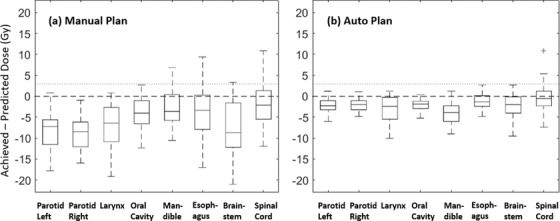
Box plots of differences between achieved and predicted doses from 25 patients with head and neck cancer in evaluation cohort 1. Panel (a) evaluates manual plans with the RapidPlan model trained with manual plans; in panel (b), the training and evaluation plans were all automated plans. Mean doses were used for analyzing left and right parotid, larynx, oral cavity, mandible, and esophagus. *D*1% doses were used for brainstem and spinal cord. Mean and *D*1% doses were derived from the upper bound of predicted dose–volume histograms (DVHs) by corresponding RapidPlan models

Figure 2 shows the dose differences for different OARs among EC1 patients. The majority of the difference values were below 0, which means that the achieved dose levels were not worse than the prediction. This was mainly because the upper bound of OAR DVH predictions were used here. With 200 structures (8 OARs for 25 patients) evaluated, 24 (12%) manual plans and 12 (6%) automated plans had worse sparing compared to prediction. When we added a margin of 3 Gy to 0 as a threshold (indicated by the dashed line in Figure 2), eight structures from the manual plans and two from the automated plans were outside the threshold. As we had matched manual and automated plans for each patient, we validated that suboptimal sparing of the eight identified structures of manual plans could be improved to reach at least the 3‐Gy threshold, as seen in the corresponding automated plans. However, the two automated plans identified with suboptimal *D*1% dose for the spinal cord could not be improved by re‐optimization without affecting other structures. This could have been caused by prediction error as outliers. Therefore, none of the automated plans should have been flagged with the 3‐Gy threshold. This threshold of dose difference plus 3 Gy was then used to flag suboptimal plans for evaluating plans from EC2.

### 3.2 Plan QA and physician review of manual test plans

**FIGURE 3:**
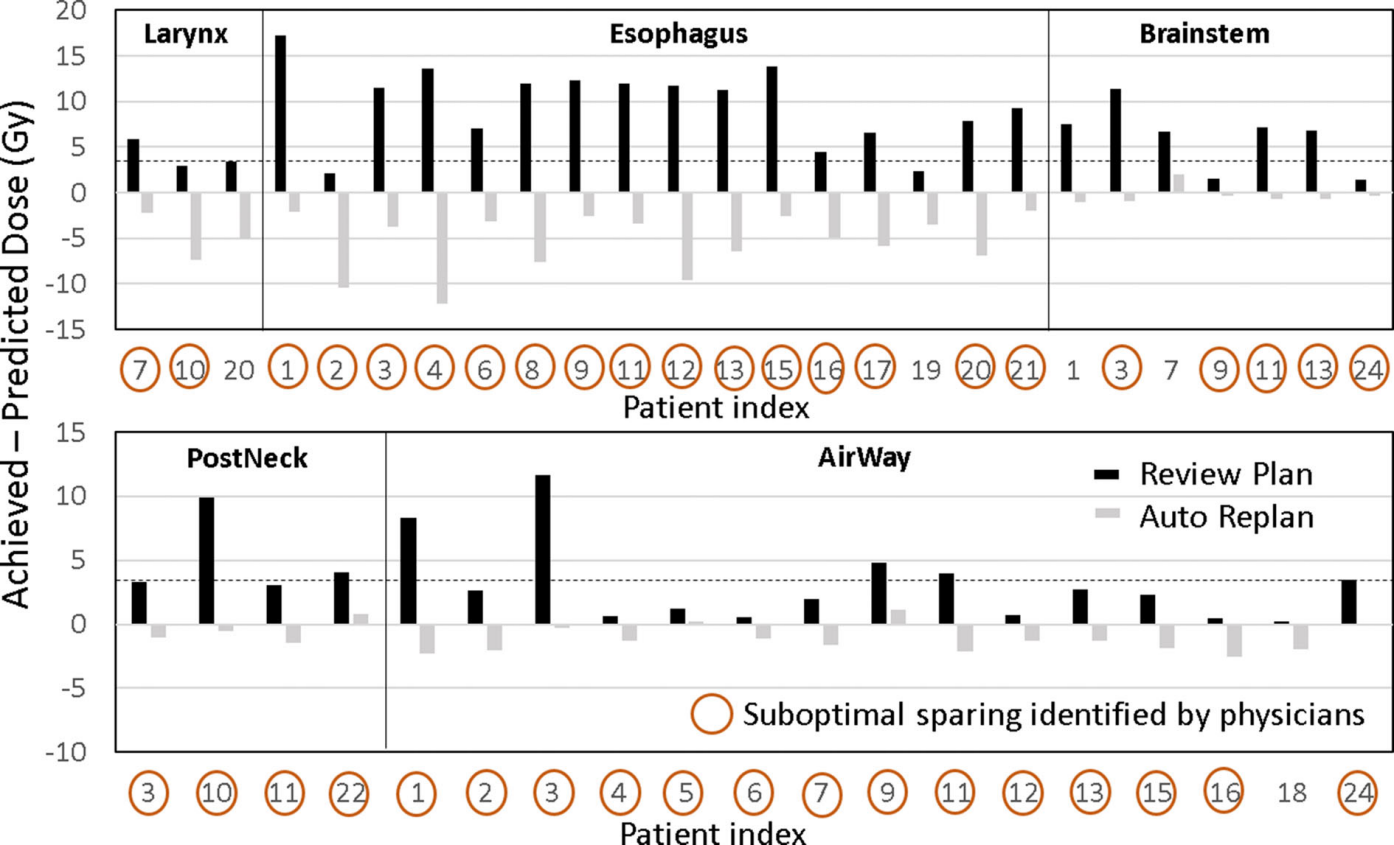
Difference between achieved and predicted mean doses for structures in suboptimal plans identified by the RapidPlan model for 25 patients with head and neck cancer. Of 45 structures with insufficient sparing based on the predictions, 40 were also identified by physician reviews

Figure 3 summarizes the plan evaluation results for the 25 patients in EC2 by the AP model. Only structures identified with lower predicted than achieved mean doses are included. Black bars indicate differences between achieved and predicted mean doses for difference structures; 45 structures (from 22 plans) were identified with positive differences, meaning possible suboptimal sparing. Note that the test plans (for EC2 patients) used in the analysis were manual plans with satisfactory dose limits but without physician review and extensive planning. Comparisons between predictions and automated plans are shown with gray bars in Figure 3. All of the identified suboptimal test plans could be improved, as shown in the regenerated automated plans. In those automated plans, 3 of the 45 structures still had higher mean doses than predictions, including 1 for brainstem, 1 for PostNeck_Avoid, and 1 for Airway_Avoid. However, the differences were all within the margin of 3 Gy as used for flagging AP plans from EC1 cohort.

Figure 3 also includes the structures identified as having possible poor sparing by physician reviews; the red circles indicate that the corresponding structures were flagged by at least one physician. For these structures, the physicians would intend to request additional planning for further improvement. The severity of overdose perceived by physicians was not considered in the plan review, so all requests from each physician were collected. Of 45 of the structures identified by the RapidPlan model, 40 (89%) were also flagged by physicians. In the post‐review discussion, physicians indicated that the five remaining structures were not flagged because they did not think further improvement was possible or because satisfactory sparing had already been achieved. All structures flagged by physicians and the RapidPlan model are included in Table S1).

## 4 DISCUSSION

In the treatment planning for complex cancer sites, such as the head and neck, with large tumor targets that are close to normal structures, it is challenging for a treatment planner to judge plan quality owing to necessary tradeoffs among various structures for individual patients. Simply meeting normal tissue dose constraints does not mean that the plan cannot be further improved. Without significant caution and time‐consuming manual interventions, inferior plans are likely to pass standard QA or peer reviews.^32^

The purpose of generating and evaluating unapproved plans in this study was to show that our automatic QA tool could detect potentially inferior plans. We attempted to use these plans to represent ones that meet dosimetric requirements but have not been optimized to reach the lowest possible doses to OARs. Then, we demonstrated the utility of this automatic tool for evaluating such plans in comparison with physician evaluations.

In addition, the complexity of radiation planning for head and neck cancer is especially impactful in low‐resource settings, in which staffing, time, and patient load can limit the ability to provide high‐quality plans for all patients. A knowledge‐based model can best predict superior dose distributions or dosimetric measures for a given anatomy only if the model is trained by superior (i.e., high‐quality) historical plans. Therefore, KBP could be a useful tool for the transfer of expert knowledge of treatment planning among hospitals and ultimately minimize the gaps in plan quality across the country and around the world.

Knowledge‐based QA does not interfere with the plan generation process and can serve as an independent tool for plan quality check, so it can be easily adopted by a clinical practice. It can be used to assist treatment planning and adjust optimization using various approaches of a planner's choice before presenting a final plan to the physician, or to provide the prediction as an additional reference to the optimized plan to support physician peer review.

As demonstrated in this study and others,^24^, ^25^ RapidPlan was effective in translating dosimetric quality of training plans into model predictions for new patients. More importantly, our data show that the model trained by automated plans was more accurate than the one trained by manual plans. For example, the predicted and achieved doses showed a stronger linear relationship for the AP model than the MP model (e.g., Figure 1). The AP model also showed higher precision than the MP model in predicting DVHs, as more consistent plan quality in OAR sparing of training AP plans than MP plans led to narrower predicted DVH band or upper‐lower DVH range (e.g., Table 3), which was determined by standard deviation and standard error of the RapidPlan regression model. In addition, for plan QA purposes, if the predicted DVH band is too wide, flagging out suboptimal or outlier plans based on such prediction would be either extremely challenging or useless. In all, these highlight the importance of using consistent and high‐quality plans in training a dose prediction model in general. Details of the auto planning approach utilized to generate AP plans in this study can be found in our previous work.^31^

It is necessary to determine a threshold of dosimetric index to distinguish suboptimal from “optimal” plans when using dose predictions for plan QA. In this study, the upper bound of the predicted mean dose was effective in evaluating the manual test plans for EC2 patients. However, an additional margin of 3 Gy added to the upper prediction was more effective in evaluating AP plans for EC1 patients because only plans that could not be improved (e.g., spinal cord in Figure 2), or false positive predictions, were found beyond this threshold. There is unlikely to be a universal threshold for different implementations of QA projects. An effective dose threshold for QA should depend on the accuracy of the prediction model and dosimetric preferences and should be determined by an evaluation study for each individual project. We should also note that using mean or max dose as a threshold alone could be insufficient to quantify plan quality especially when specific dose–volume limits to OARs are prescribed. Moreover, using the same recipe of threshold, for example, upper prediction or an additional margin, for all OARs may also lead to flagging false positive or false negative plans. Therefore, developing more flexible and comprehensive approaches to flagging suboptimal plans are important directions in our future work, such as including dose–volume indices, weighted plan quality score (e.g., Plan Quality Metric^33^), structure‐specific threshold, etc., in future models.

The inclusion of the two planning structures in the DVH predictions is unique to our study. We have learned from our previous experience that these structures are important to control and measure overdose to normal tissues in the back of the neck (PostNeck_Avoid) and the swallowing structures between two lateral PTVs (Airway_Avoid) in many head and neck VMAT plans.^31^ This practice was successfully reflected in the RapidPlan model trained by automated plans and used for QA of new test plans (EC2) with good agreement with physician reviews (Figure 3). This study demonstrated that RapidPlan can predict DVHs not only for anatomical structures but also for non‐anatomical ones that are useful in evaluating plan quality.

While physician reviews largely concurred with the knowledge‐based QA results (Figure 3), 23 of 63 structures flagged by physicians were not identified by the RapidPlan models (Table S1). This highlights the complexity of plan quality evaluation, both manual and automated. First, automatic QA can provide a useful tool to assist plan evaluation, but still needs manual approval by the treatment planner or physician. For example, in one of the three cases, where the spinal cord was flagged by physicians but not RapidPlan, the regenerated auto plan could reduce maximum spinal cord dose to the model predicted range. This was caused by inaccurate DVH prediction for this case. The same happened for one of the cases where the oral cavity was flagged by physicians. Second, variability in physicians’ preferences for plan quality and clinical practices also exists. For example, one physician did not favor certain overdosing to the mandible in a few plans, but those plans were not detected by the automated QA. This discrepancy was essentially caused by different practices among institutions, as our model training plans sometimes included part of the mandible in PTVs, which was discouraged at this physician's home institution. Third, a physician's judgment of plan quality may favor exceptional protection of certain normal tissues but may allow slightly higher doses to structures that had already achieved adequate sparing. Therefore, automatic QA may allow these scenarios to be identified in clinical practice, highlighting what could be improved (if desired). Importantly, we may also note that automatic QA is not aimed to replace manual or physician review in which patient medical evaluation is considered comprehensively and physician preferences are enabled. It is possible to build KBP models per physician, which is an open question currently. In all, the goal of the present work should be facilitating plan review to improve its efficiency and helping identify possible suboptimal sparing that might be overlooked in manual review.

One limitation of this study is to only include a subset of OARs in head and neck radiotherapy and only mean and maximum dose metrics for model generation and evaluation. However, there are many other critical structures such as submandibular glands, brachial plexuses, optic nerves, etc., as well as more descriptive dose‐volume metrics to measure plan quality. Based on the present proof of concept study, we will expand our model and analysis in our future works. Another limitation of RapidPlan DVH predictions is that target DVHs cannot be modeled and predicted. Thus, the user's judgment on satisfactory dosimetry for targets, for example, coverage, homogeneity and conformity, is required when assessing predicted OAR DVHs. In the present study, we only ensured that target doses of the evaluation plans met our clinical standards. This could be remedied by using methods that can predict DVH for both target volumes and OARs.^34^, ^35^ Recent studies have also proposed methods to predict voxel‐based 3D dose distributions,^36^, ^37^, ^38^ which are additional promising tools for plan quality control and assurance. Our future work may include building RapidPlan models from reoptimized plans according to multiple physician reviews such as ones collected in this study. Furthermore, future KBP solutions can also extend plan quality measures such as robustness, plan complexity, and delivery efficiency,^39^ in addition to dosimetric indices, in prediction models.

## 5 CONCLUSION

Our study shows that knowledge‐based DVH prediction models can be sufficiently accurate for plan QA purposes. A RapidPlan model trained by a small cohort of automated plans was effective for detecting suboptimal plans for patients with head and neck cancer. Re‐generated automated plans validated that OAR sparing of those detected plans could be further improved. While the majority of the suboptimal plans identified by DVH prediction were in agreement with physicians’ plan review, we also observed variability in plan quality preference among physicians. Nevertheless, accurate DVH prediction models have potential for improving consistency and efficiency of the plan QA workflow for individual patients in the clinic.

## AUTHORS’ CONTRIBUTION

Wenhua Cao and Laurence E. Court designed the study. Wenhua Cao performed the data collection and analysis, wrote the manuscript. Mary Gronberg and Adenike Olanrewaju assisted in data collection and revising the manuscript. Thomas J. Whitaker, Karen Hoffman, Carlos Cardenas, Adam S. Garden, Heath D. Skinner, and Beth M. Beadle provided critical physics and clinical inputs to the study and manuscript. Beth M. Beadle and Laurence E. Court helped to revise the manuscript. All authors contributed to this study and approved the submitted manuscript.

## CONFLICT OF INTEREST

This project was performed by the Radiation Planning Team at MD Anderson, which receives funding from the NCI, CPRIT, Wellcome Trust and Varian Medical Systems.

## Supporting information

Supporting Information

## Data Availability

The access to patient data including clinical treatments and images used in this study are restricted by the University of Texas MD Anderson Cancer Center.
