# Supplementary material for: Knowledge‐based planning for the radiation therapy treatment plan quality assurance for patients with head and neck cancer
Source: J Appl Clin Med Phys. 2022 Apr 30;23(6):e13614. doi: 10.1002/acm2.13614 (PMC9195018; doi:10.1002/acm2.13614)
Supplement: Supplementary file 1 — Supporting Information [file ACM2-23-e13614-s001.docx]

**Supplementary Materials**


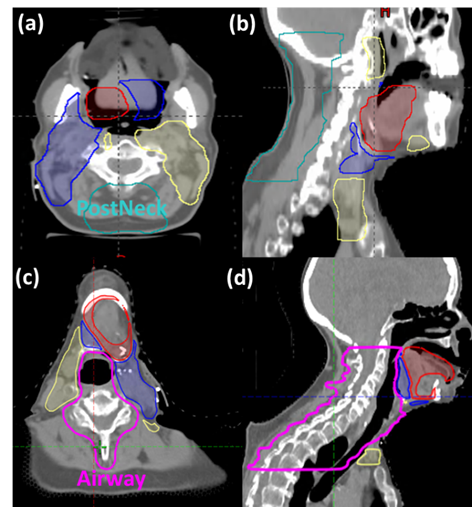


Figure S1. Example of two normal tissue avoidance structures used in automated treatment planning: PostNeck_Avoid (a-b) and Airway_Avoid (c-d). Contours in red, blue and yellow indicate PTVs with high, medium, and low prescription doses, respectively.


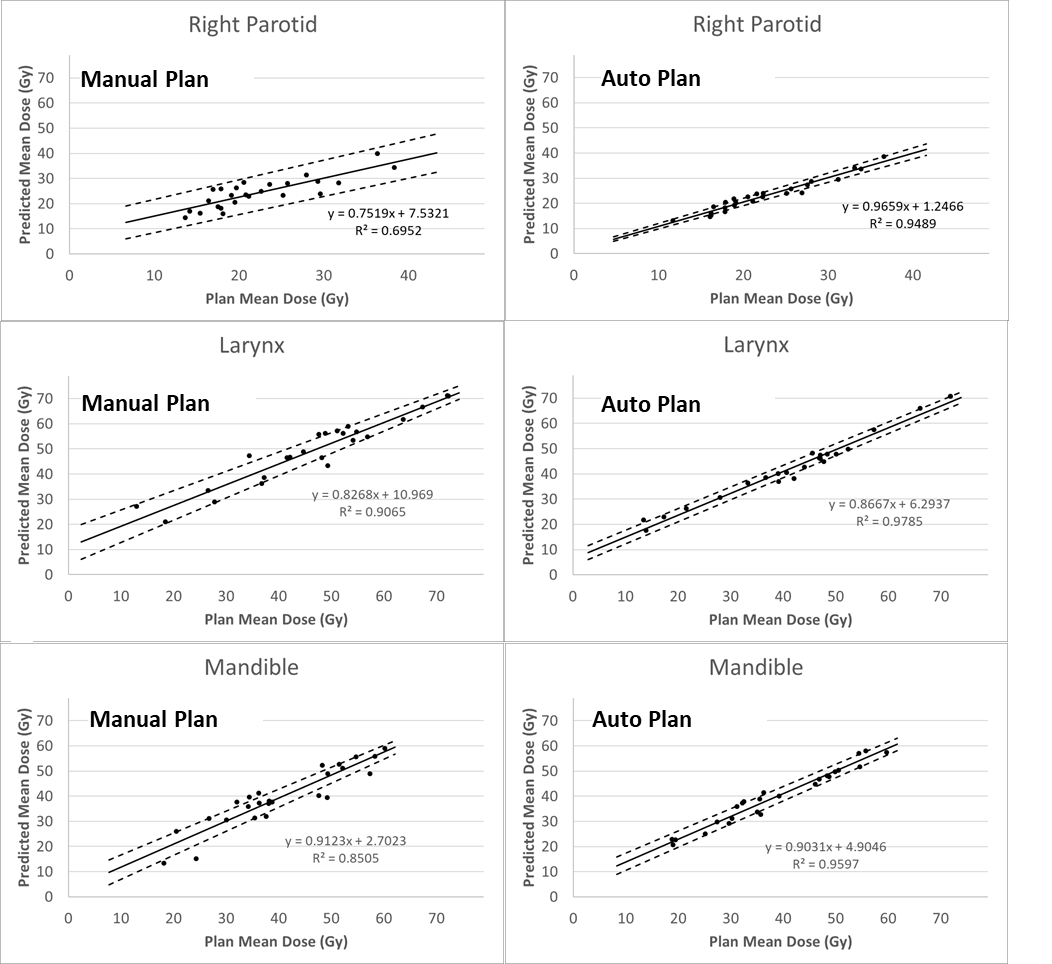


Figure S2. Linear regression between achieved and predicted mean doses of left parotid, oral cavity, and esophagus for 25 patients in Evaluation Cohort 1. Solid lines are fitted by achieved mean dose of manual or automated plan and predicted mean dose (derived from the average between upper and lower predicted DVHs by RapidPlan) of MP or AP model. Dashed lines indicate regressions using upper and lower predictions.

Table S1. Structures identified with suboptimal sparing by physician review (x) and RapidPlan QA (Δ).

Note: Multiple x indicates that the corresponding structure was flagged by multiple physicians.

| Patient | Parotids | Larynx | O. Cavity | Mandible | Esophagus | Brainstem | Cord | PostNeck_Avoid | AirWay_Avoid |
| --- | --- | --- | --- | --- | --- | --- | --- | --- | --- |
| 1 |  | x |  |  | xΔ | Δ |  |  | xΔ |
| 2 | x |  | xx | x | xΔ | x |  | x | xΔ |
| 3 | x |  |  |  | xxΔ | xxxΔ | xx | xΔ | xxΔ |
| 4 |  |  |  |  | xΔ |  |  | x | xΔ |
| 5 |  |  |  |  |  |  |  |  | xΔ |
| 6 |  |  |  | x | xxΔ |  |  |  | xxΔ |
| 7 |  | xΔ |  |  |  | Δ |  | x | xΔ |
| 8 |  |  |  |  | xxΔ |  | x |  |  |
| 9 |  |  |  | x | xΔ | xΔ |  |  | xΔ |
| 10 |  | xΔ | x | x |  |  |  | xΔ |  |
| 11 |  |  |  | x | xΔ | xΔ | x | xΔ | xΔ |
| 12 |  |  |  |  | xxΔ |  |  | x | xxΔ |
| 13 |  |  |  |  | xΔ | xΔ |  |  | xΔ |
| 14 |  |  | x |  |  |  |  |  |  |
| 15 |  | x |  |  | xxΔ |  |  |  | xxΔ |
| 16 |  |  |  |  | xΔ |  |  |  | xΔ |
| 17 |  | xx |  |  | xxΔ |  |  | x | x |
| 18 |  |  |  |  |  |  |  |  | Δ |
| 19 |  |  |  |  | Δ |  |  |  |  |
| 20 |  | Δ |  |  | xΔ |  |  | x |  |
| 21 |  |  |  |  | xΔ |  |  |  |  |
| 22 |  |  |  |  |  |  |  | xxΔ |  |
| 23 |  |  |  |  |  |  |  |  |  |
| 24 |  |  |  | x |  | xΔ |  |  | xxΔ |
| 25 |  |  |  |  |  |  |  | x |  |
